# Menopause knowledge and education in women under 40: Results from an online survey

**DOI:** 10.1177/17455057221139660

**Published:** 2022-12-19

**Authors:** Carly Munn, Leigh Vaughan, Vikram Talaulikar, Melanie C Davies, Joyce C Harper

**Affiliations:** 1 EGA Institute for Women’s Health, University College London, London, UK; 2 Reproductive Medicine Unit, University College Hospital, London, UK

**Keywords:** education, menopause education, menopause symptoms, menopause, perimenopause

## Abstract

**Background:**

All women experience the menopause, yet education around the topic is limited. Studies conducted in women aged over 40 show that women have limited knowledge about the menopause.

**Objectives:**

This study aims to understand what women under 40 know about the menopause, how they have acquired this knowledge and where they think menopause education should be taught. This data will help to determine how to effectively deliver menopause education.

**Design:**

A survey was designed that asked women under 40 what they know of, and their attitudes to, the menopause, using Qualtrics XM software.

**Methods:**

The survey was advertised for 5 weeks on social media. Six questions related to menopause education were analysed. Responses between age groups under 20, 21–30 and over 30 were compared using a chi-square test. A thematic-style analysis was also conducted on a free-text question where answers referred to education.

**Results:**

A total of 738 women’s responses were included in the analysis; over 80% had no knowledge or just some knowledge of the menopause. Women over 30 used official websites (p = 0.017) and scientific literature (p = 0.047) significantly more than other age groups to learn about the menopause, while women under 20 were more likely to learn from family members (p = 0.002). These women felt education should start in schools.

**Conclusion:**

Many women under 40 have limited education of the menopause. Women under 20 are more passive in their approach to learning about the menopause compared with those over 30, who are more proactive. Menopause education strategies must start at school and extend beyond schools adopting a multifaceted approach; it is recommended that the workplace, social media and public health campaigns are used to deliver menopause education moving forward.

## Introduction

Menopause, as defined by the World Health Organization (WHO),^1^ is the permanent cessation of menstrual cycles for 12 months. The perimenopause is a period of transition during which women experience dynamic changes in their physical, emotional and mental health. Globally, in high-income countries, the age at which most women experience menopause is between 49 and 52.^2^

Historically, there has been little to no teaching of the menopause, leaving generations of women with a detrimental knowledge gap. But in the United Kingdom in the past decade, significant progress surrounding menopause education has been made. In 2015, the first National Institute for Health and Care Excellence (NICE) guidelines were published for menopause,^3^ reflecting the growing acceptance of its importance in healthcare. Further to this, in 2019 teaching of the menopause became compulsory in schools as part of Relations and Sex Education (RSE).^4^

However, there is an education gap. We conducted a survey in women over age 40 and found that perimenopausal women are overwhelmingly uneducated on the menopause and angry that they had to experience it with such little knowledge.^5^ In a study of women over 40 it was found that women’s (limited) knowledge came mainly from relatives (26.8%) and friends (25.5%) and not formal education sources.^6^ Links between women’s knowledge of the menopause and their education, socioeconomic status and employment,^7^ and ethnicity^8^ have been shown.

There is limited research conducted into younger women’s education and knowledge of the menopause. One study conducted in Egypt with women of reproductive age showed that they had very limited knowledge of menopause^9^ and another, carried out in Jordan, found that although women had knowledge of the menopausal concept, there was little awareness of menopausal consequences.^10^ Our study aims to understand the current climate of menopause knowledge in women under 40 while simultaneously gaining an insight into how they have acquired this knowledge. Through doing so, we hope to determine how to deliver menopause education.

## Methods

This was a mixed-methods, observational study. The article follows the Strengthening the Reporting of Observational Studies in Epidemiology (STROBE) guidelines.

The survey was approved by the UCL Research Ethics Committee ID no: 9831/005.

Qualtrics XM was used to create our online survey compiled of 35 questions (Supplemental data). Our previously published survey for women over 40 was taken as a template^5^ and was edited by the research team; any questions that were irrelevant to women under 40 were deleted.

The survey started with background information and the UCL Data Protection Privacy Notice to ensure participants understood that their answers were anonymous. Consent was obtained by a question embedded in the survey, ensuring participants understood the eligibility criteria and reminding them they were able to withdraw at any point prior to submitting their answers.

The survey was split into three parts: (1) information on the participant, including their country of residence, age, sexual orientation, relationship status, number of children, well-being and contraceptive methods, (2) the main body of research questions which focused on participants’ experience and learning of the menopause. There were 13 questions in this section, of which 12 were closed in nature with multiple-answer options and 1 was a free-text question, ‘In your own words, would you like to tell us anything about your views of the perimenopause/menopause?’ (3) Background information on the participants including their highest educational qualification, profession, religion, ethnicity and disability status. To ensure women did not feel pressured to give information, all demographic questions throughout the survey included an answer option reading ‘prefer not to say’.

The inclusion criteria were any woman under age 40 who could read written English and had not gone through an early menopause. The exclusion criteria were any woman over age 40.

The survey was validated by undertaking eight cognitive interviews on women fitting the inclusion criteria. This was done primarily to ensure the survey’s wording and questions were clear, but it also gave an overview of how the survey generally flowed for the reader. Researchers sat with interviewees while they filled out the survey, documenting any verbal feedback provided during the process.

The survey was launched on multiple social platforms where it was live from 8 February to 15 March 2022. We initially released the survey on the Global Women Connected Facebook page (run by Professor Joyce Harper) and then extended this to the personal social media pages of the three researchers involved in the project: Joyce Harper, Leigh Vaughan and Carly Munn. This included Twitter, Instagram, LinkedIn and Facebook pages. From 2 to 5 March, a paid Twitter advert was used which assisted in collecting responses.

### Data analysis

This article focused on menopause education in women under 40 and therefore analysed the following questions of the survey:

How informed do you feel about the perimenopause/menopause?
How were you taught about the menopause at school?
Have you looked for information or discussed the menopause?
Where have you looked for this information?
Do you feel there is an open discussion with friends/family about the menopause?
Where do you think the menopause should be taught?

The data were analysed in three groups specified by age: under 20, 21–30 and over 30.

Statistical analysis was performed using SPSS and the chi-square test to determine whether the differences between age groups were statistically significant. Results were considered statistically significant if the p value generated was less than 0.05. Those results that were statistically significant are marked with an asterisk on the relevant graph and the p value included in the text.

The free-text question asked women to tell us about their views on the perimenopause/menopause; responses were examined to see whether they mentioned education and were analysed thematically.

We had aimed for around 1000 women to ensure saturation.

## Results

There were 870 responses to the survey but 120 did not complete the survey and 12 participants were over 40; these were excluded, and 738 responses were analysed.

### Sociodemographic characteristics

The demographics of the 738 participants are shown in Table 1. Most participants were in the age range of 21–30 (49.1%, 362/738). The average age of participants was 27. The majority of participants were from the United Kingdom (93.1%, 687/738) and most were heterosexual (72.2%, 533/738). Participants predominantly did not have children (77.2%, 570/738) and their highest educational qualification was either University undergraduate (38.8%, 286/738) or University postgraduate (36.0%, 266/738). Most women were of White – English/Welsh/Scottish/Northern Irish/British ethnicity (80.2%, 592/738).

**Table 1.** The demographics of the women who completed the survey.

Age	n	Frequency (%)
Under 20	100	13.6
21–30	362	49.1
31–40	276	37.4
Country of residence		0.00%
UK	687	93.1
Other	51	6.9
Sexual orientation		0.00%
Heterosexual	533	72.2
Homosexual	30	4.1
Bisexual	137	18.6
Pansexual	13	1.8
Asexual	11	1.5
Prefer not to say	14	1.9
Relationship status		0.00%
Single	217	29.4
In a relationship not cohabiting	135	18.3
In a relationship cohabiting	169	22.9
Married/civil partnership	209	28.3
Prefer not to say	3	0.4
Other – in your own words	5	0.7
Widowed	0	0.0
Parity		0.00%
1	77	10.4
2	74	10.0
3	10	1.4
4 or more	3	0.4
I do not have children	570	77.2
Prefer not to say	4	0.5
Highest Educational Qualification		0.00%
Secondary school	45	6.1
University undergraduate	286	38.8
University postgraduate	266	36.0
Other	9	1.2
Prefer not to say	3	0.4
A level/college-level	129	17.5
Ethnicity		0.00%
White-English/Welsh/Scottish/Northern Irish/British	592	80.2
White-Irish	33	4.5
Any other White background	74	10.0
Black/Black British-African	11	1.5
Any other Asian background	8	1.1
Mixed ethnic background	18	2.4
Arab	1	0.1
Any other ethnic group	4	0.5
Black/Black British–Caribbean	1	0.1
Any other Black/African/Caribbean background	1	0.1
Asian/Asian British–Indian	11	1.5
Asian/Asian British–Pakistani	1	0.1
Prefer not to say	3	0.4
Latino	6	0.8

#### Knowledge of menopause

**Figure 1.**
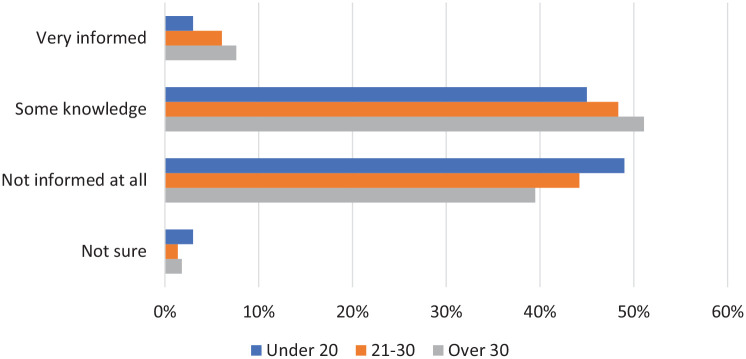
Women were asked how informed they feel about the menopause.

Women were asked how informed they felt about the menopause (Figure 1). The options offered to participants were ‘very informed’, ‘some knowledge’, ‘not informed at all’ and ‘not sure’. Across all age groups the most common feeling was that they were not informed at all or had some knowledge of the menopause; less than 10% felt very informed. There was no significant difference between the groups (p = 0.39).

#### Menopause education

**Figure 2.**
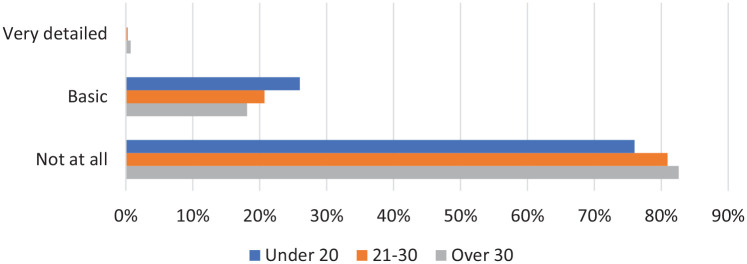
Women were asked how they were taught about the menopause at school.

We asked women how they were taught about the menopause at school (Figure 2). Participants were offered three options ‘very detailed’, ‘basic’ and ‘not at all’. The vast majority were not taught about the menopause at all. Fewer than 1% of women across all age groups were given very detailed teaching on the menopause. Basic teaching was most common in women under 20 (26.0%, 26/100). Differences between age groups were not statistically significant (p = 0.561).

**Figure 3.**
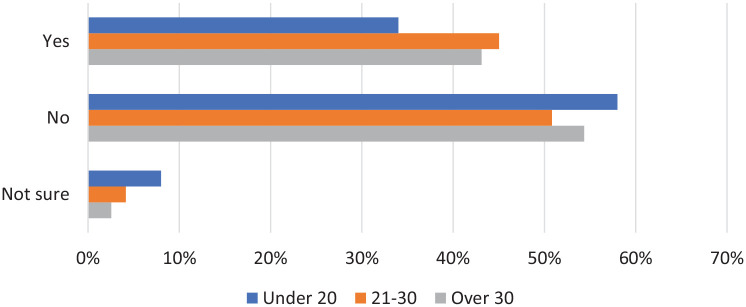
Women were asked if they had looked for information or discussed the menopause.

With the answer options ‘yes’, ‘no’ and ‘not sure’ we asked women if they have looked for information on the menopause. Over 50% of women in all age groups answered no (Figure 3). Women over 30 (43.1%, 119/276) and aged 21–30 (45.0%, 163/362) were most likely to have looked for information on the menopause. The difference between age groups was not statistically significant (p = 0.077).

**Figure 4.**
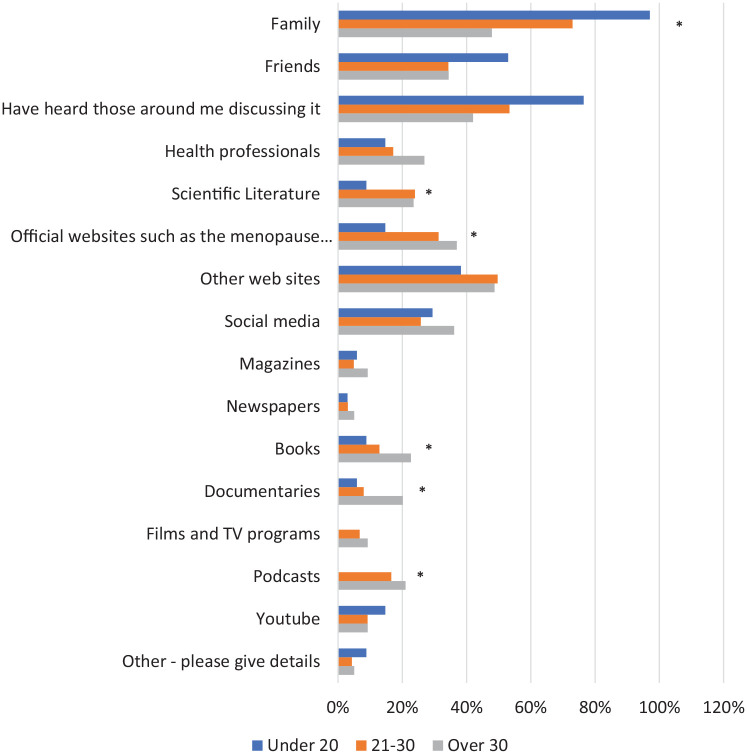
Women were asked how they had looked for information on the menopause. *Significant result.

Family was the most common source of information on the menopause (Figure 4) for women under 20 (97.1%, 33/34) and 21–30 (73.0%, 119/163); however, this was lower for women over 30 (47.9%, 57/119). This difference between age groups was statistically significant (p = 0.002). Official websites (p = 0.017), scientific literature (p = 0.047), podcasts (p = 0.008), documentaries (p = 0.005) and books (p = 0.032) were used by women over 30 or aged 21–30 more than those under 20, and these differences were also statistically significant. Social media was used fairly consistently across age groups, with around 30% of women learning in this way. The option ‘Other’ was selected by 2%–3% of women in each age group and this option was followed by a space for free text. Within this, multiple women aged 21–30 or over 30 mentioned they had been given information on the menopause through work. Others had proactively looked for it via Google and the National Health Services (NHS) website. In women under 20, sources of information included period tracker apps and teachers.

**Figure 5.**
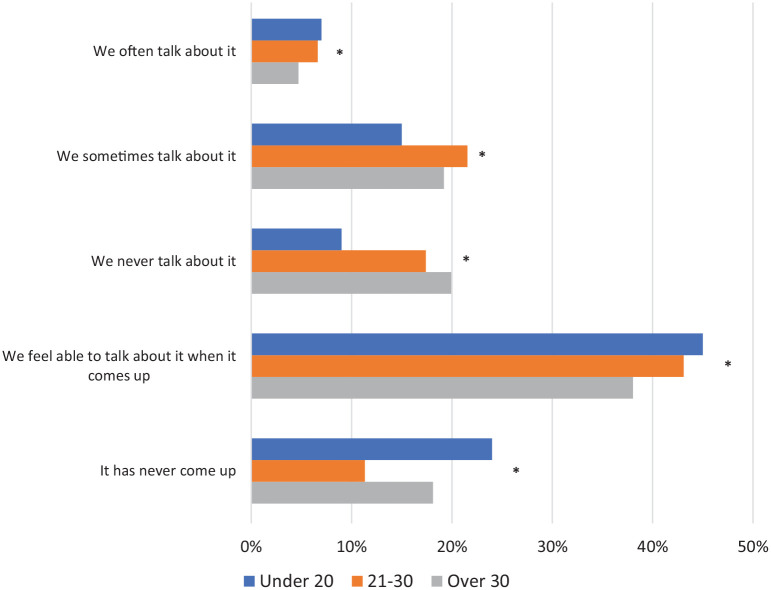
Women were asked whether they feel there is an open discussion with friends/family about the menopause. *Significant result.

We asked women if they feel there is an open discussion with friends/family about the menopause (Figure 5). The answer options offered to participants were ‘we often talk about it’, ‘we sometimes talk about it’, ‘we never talk about it’, ‘we feel able to talk about it when it comes up’ and ‘it never comes up’. Across all age groups, under 10% of women said they often talk about the menopause and around 40% said they feel able to talk about it when it comes up. Women over 30 were the most likely to often talk about the menopause (6.6%, 13/276). In women under 20, 24.0% (24/100) said that the menopause has never come up in their conversations. The observed differences between age groups for all responses to this question were statistically significant (p = 0.011).

## Where should the menopause be taught?

**Figure 6.**
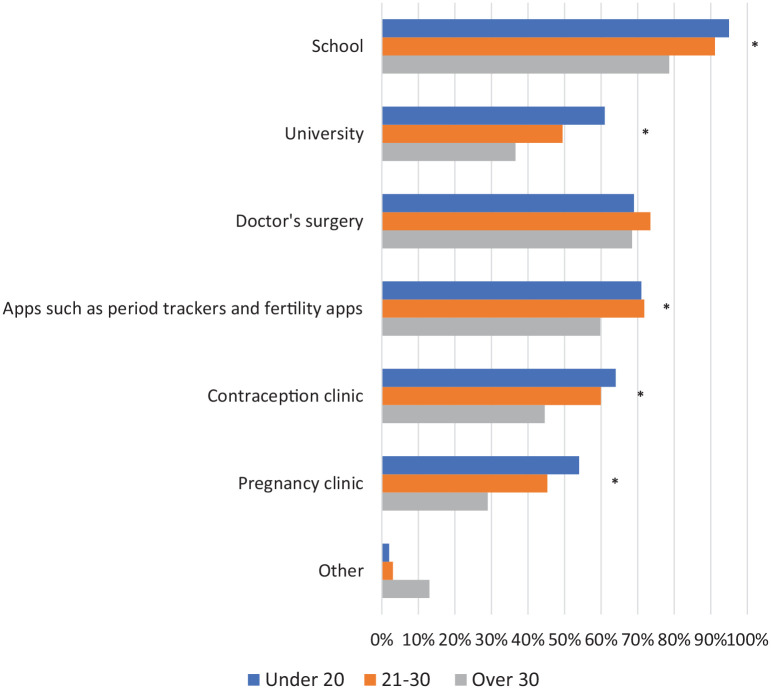
Women were asked where they think the menopause should be taught.

We asked women where they think the menopause should be taught (Figure 6). Participants were offered seven different answer options for this question, one of which was ‘other’. The majority of women thought that the best place for teaching was at school, with women under 20 (95.0%, 95/100) or aged 21–30 (91.2%, 330/362) most likely to select this option; differences between age groups were statistically significant (p < 0.001). Women under 20 and aged 21–30 were also more likely to feel apps are an appropriate place for menopause education than women over 30; this difference was also statistically significant (p = 0.004). Other observed differences between age groups that were statistically significant were for university, pregnancy clinics and contraceptive clinics; women under 20 were most likely to have selected these options (p = 0.001). The option ‘Other’ was selected by women over 30 the most (13.0%, 36/276), this difference between age groups was statistically significant (p = 0.001). For those who selected ‘Other’, a space for free text followed. The most popular suggestion proposed, particularly by women aged 21–30 or over 30, was the idea of education within the workplace or through online formats, including webinars and online courses such as Eventbrite. Furthermore, many felt public health campaigns, including TV adverts and social media advertising, would be good methods to deliver education. Education in a more formal setting such as in pharmacies, sexual health clinics or the setting up of public health menopause clinics were also suggested. The necessity of thorough menopause education throughout medical school and further into medical careers was also highlighted.

## Qualitative comments about menopause education

In a free-text question, women were able to tell us about their views of the perimenopause/menopause. Around 1/3 of women in each age group submitted an answer; however, engagement with this question was highest in women over 30 (40.0%, 110/276). Answers from all participants were reviewed and those that referred to education were isolated, so a thematic analysis could be conducted; this included the identification, analysis and reporting of common themes. The most prevalent themes found among these answers were a willingness to learn, frustration at the lack of education, the importance of teaching men, where the menopause should be taught, watching others go through it and health professionals’ lack of knowledge.

### Willingness to learn

A key theme in the answers from all three age groups was a willingness to learn. Almost all the women said that they would like to or ‘wished’ they knew more. For many of these women, the desire to be educated stemmed from the fact it would make them less ‘scared’ and more comfortable if they knew about what was to come:

I would love for menopause education to be more detailed and definitely more wide-spread. I am 26 but would feel much more comfortable if I felt informed about what may be to come. (#593, age 26)

I am not well informed to know about how I feel about menopause, but am open to learning about it. (#33, age 23)

### Frustration at the lack of education

Frustration at their lack of education was also a key part of women’s answers, particularly those over 30, with one woman feeling ‘furious’ she had not received any teaching on the menopause. Many framed this frustration within the context that menopause education is essential because it is a ‘key part of half the population’s lives’:

I am furious I was not told anything about the menopause and had not even heard the word perimenopause. (#453, age 39)

I wish it was part of school curriculum. It feels insulting that it’s not, given that it’s such a key part of half the population’s lives. (#707, age 24)

### The importance of teaching men

Not only were women keen to learn more about the menopause, but they also felt it was essential that men were educated on it too; this was observed across all age groups. Reasoning for this varied, but the most common feeling was a desire for universal understanding of the menopause so that men become ‘less ignorant’ and can help support women through this life stage:

It should be taught in schools and NOT just to females – everyone should know – this will reduce some of the ignorance or males. (#592, age 27)

Education needs to be not just women who will experience it, but boys at school also so they can grow into well informed men who can support their partners and understand when their mothers may be going through it. (#588, age 32)

### Where should the menopause be taught

Women’s views on where the menopause should be taught varied. Some, especially those aged 21–30 and over 30, felt school was too young and thus children may be disengaged. Others disagreed and felt it was entirely appropriate. Many women recognized that school is a unique and convenient opportunity for education of the whole population and therefore may be a good place to teach about the menopause:

I’m not sure school is the right place to prepare for it, as it feels so far away that I’m not sure people would be fully engaged. (#105, age 35)

I wish it was taught earlier in life in convenient settings i.e. school, rather than having to go to a doctor to find out the information. (#195, age 22)

A mention of the workplace as an appropriate place for menopause education was also frequent, particularly in women aged 21–30 and over 30. One woman felt that a lack of education among employers contributes to the stigma around the menopause:

I think the workplace has a strong need to talk more and educate more on this. (#102, age 36)

Employers should have better understanding. So that it becomes less taboo or something to hide or feel ashamed of. (#409, age 36)

The suggestion of menopause education via public health campaigns was also suggested by women, particularly those over 30:

Through public health messages in same way as e.g. healthy diet. (#18, age 40)

I think a sustained public health campaign would be of significant benefit. (#344, age 35)

### Watching others go through it

Most women, especially those under 20 and aged 21–30, had gained their (limited) knowledge of the menopause from watching others go through it, most commonly their mothers. One woman described her learning in this way as ‘indirect’. Multiple women felt that any attempt to gain knowledge beyond this was restricted as information on the menopause is difficult to access and unreliable:

My only knowledge comes from my mother’s experience of menopause. (#787, age 34)

I don’t really know much about it other than what I’ve indirectly gathered from people I know who’ve been through it. (#507, age 23)

Education about is lacking and misinformation online is even worse! (#100, age 24)

### Health professionals’ lack of knowledge

The issue of health professionals not having enough knowledge on the menopause was particularly mentioned by women over 30. This was mostly highlighted through anecdotes of misdiagnosis, both their own and/or that of someone they knew. Furthermore, feelings that misinformation deters women from seeking help from health professionals in the first place were also prevalent:

I feel that a lot of health care professionals don’t have adequate training in the menopause. (#675, age 39)

I was missed. I have been back and forward to the GP with most of the symptoms above for some time, but because I was still having periods. I was dismissed. I was told all my bloods and thyroid were fine (no bloods taken to check hormone) I was told to take anti depressants, beta blockers and to refer myself in to mental health services. I knew this wasn’t right. I eventually had to stamp my foot and demand bloods, only to be told I was menopausal, POF (premature ovarian failure) at age 39. GP’s and health professionals are not educated enough on peri/menopause. (#251, age 30)

## Discussion

One aim of our study was to understand what women under 40 know about the menopause and how they have acquired this knowledge. A second aim was to gain an insight into how they think menopause education should be delivered moving forward. Understanding how women under 40 learn, and want to learn, about the menopause is necessary so that education strategies can be correctly focused moving forward. Ultimately this will improve menopause education and thus increase knowledge among future generations.

## Menopause knowledge

Our study shows that women under 40 have a significant lack of knowledge surrounding the menopause. Over 80% of women under 40, across all age groups, either have ‘no knowledge at all’ or just ‘some knowledge’ of the menopause. Women over 30 were most likely to feel ‘very informed’ about the menopause; however, this was still a very small proportion (7.6%). Although our study is the first to identify the significant knowledge gap surrounding the menopause that exists in women under 40, similar outcomes have been observed in women over 40.^5,11^ Menopause knowledge in older women appears to be equally limited,^11^ and a distinctive emotion observed in these older women was anger at this lack of education;^5^ anger was less prevalent in women under 40. Research conducted to curate ‘The Women’s Health Strategy for England’ provides further evidence for this knowledge gap and suggests it can exist right up until women are experiencing the menopause.^12^

## Menopause education

Our findings demonstrate that in women under 40, over 50% across all age groups have not looked for any information on the menopause. This clearly shows that there is not a consistent interest in the menopause among young women. Women aged 21–30 or over 30 were most likely to have looked for information on the menopause, and the most common way this was done by women across all age groups was using family and friends, discussion with those around them and official websites. Very few women have learnt about the menopause via more formal methods such as school.

Most women under 40 have not been taught about the menopause at school; around 80% of women aged 21–30 (80.9%) or over 30 (82.6%) received no teaching in this environment at all. The updated 2019 Sex and Relationships Education Regulations that made menopause education in schools compulsory^4^ aims to change these figures, and our study may reflect the beginnings of the effect of this act; women under 20 were most likely to have received basic menopause education at school (26.0%). However, our data also show that three-quarters of women under 20 have not received any teaching of the menopause at school and therefore clearly there will be a time lag before the new regulations are fully effective. The 2019 Sex and Relations Educationship Regulations specifies that all state-funded schools following the national curriculum must deliver teaching on the menopause as part of Personal Social Health and Economic Education (PSHE).^4^ Despite this, the way in which this teaching should be delivered, and further what should be included within this education, is not specified. This admits some space for inconsistent teaching between schools, a risk that could be minimized with more extensive resource provision.

With a lack of formal education sources, a significant proportion of women under 40 have undertaken self-directed learning of the menopause; this was most common in women aged 21–30 and over 30. Closer analysis of how women under 40 had approached this self-directed learning revealed differences between age groups; women over 30 were much more proactive in their approach to finding information, while women under 20 used far more passive routes to acquire information on the menopause.

Women over 30 were more likely than those under 20 to take a proactive approach to learning about the menopause. Although they used family and friends, these women relied on official websites, social media and scientific literature as sources of information more than women under 20. The use of scientific literature was higher in women over 30 with statistical significance. As women are unlikely to interact with these resources incidentally, and more likely to be proactively seeking them, women over 30 appear to have a stronger desire to learn about the menopause than those under 20. This may be explained by the fact that younger women simply have not thought about the topic as they are further away from menopausal age. In identifying these patterns, education strategies aimed at women over 30 require less focus on stimulating interest in the menopause and instead must prioritize ensuring the resources women are proactively seeking are clearly signposted, easily accessible and contain valid information. Multiple studies conducted in women over 40 have shown that older women take a similar proactive approach to learning about the menopause, using websites such as The British Menopause Society (BMS),^5^ newspapers/magazines^13,14^ and doctors/health professionals.^6,15^

Women under 20 and aged 21–30 were more likely to use passive methods to learn about the menopause; family, friends and hearing those around them discussing the menopause acted as their main methods of learning. The use of family, in particular, was significantly higher in younger women than those over 30. These findings suggest that younger women are not actively looking to learn about the menopause but are acquiring knowledge on the topic incidentally, through interacting and engaging with it in their day-to-day lives. Multiple studies conducted in women over 40 have also found friends and family to be women’s main source of menopause knowledge,^6,15^ and the importance of family for health education promotion is recognized by studies.^16^

Although our study demonstrates that younger women mainly learn via friends and family, we also found that these learning opportunities are sparse; 24% of women under 20 have never been in a conversation with friends and family in which the menopause has come up. Similar trends were observed in women aged 21–30 or over 30, where around 20% of women said they never talk about the menopause. This lack of discussion has been explored more widely, in books such as *The Silent Passage*^17^ and in literature,^18^ where one paper refers to the menopause as an ‘intergenerational silence’.^19^ A US study conducted in women over 40 has also demonstrated similar trends, with 30% of women saying they have never spoken to anyone about the menopause, including friends and family.^20^

Recognizing that menopause discussion among friends and family is infrequent, yet it acts as an important education source for young women, shows that education strategies for these age groups must prioritize promoting and facilitating more frequent discussion around the menopause. Ultimately, this will give women more opportunities to interact with the topic day-to-day and acquire knowledge.

## Solutions

The delivery of effective menopause education moving forward is essential. Our study shows the impact a lack of education and thus a lack of discussion is having; many women feel ‘scared’ about the menopause and one woman recognized that a lack of knowledge is significantly perpetuating the ‘taboo’ attached to the menopause. Multiple studies have shown similar ideas, acknowledging that a combination of silence and a lack of education greatly perpetuates the stigma that exists around the menopause.^14,19,21,22^ Further benefits of effective menopause education have been suggested by other studies; it will make it easier for women to identify symptoms and seek treatment^23,24^ and may improve their positive attitude towards the menopause.^25^

This study shows that most women under 40 have not been educated on the menopause at school. Despite this, these women do recognize that school should be the place to educate future generations; over 75% of women across all age groups said they thought the menopause should be taught at school. Within our study, multiple recommendations were made by women surrounding how they think menopause education in schools can be best delivered. Women under 40 were emphatic that education of young men on the menopause is equally as essential as the education of young women. This was also observed in the study with women over 40.^5^ Ultimately, education of both men and women is essential to create a society that universally understands and has an awareness of the menopause. It was also clear that a key reason young women want to learn about the menopause is so that they can support family or friends going through it. Education at school must therefore include advice on how to help others experiencing the menopause and encourage open discussion about the menopause between pupils and those around them. Finally, as formerly explored, young women appear to be more passive in their approach to gaining information on the menopause. Although school greatly aligns with this method of learning (by informing young people without them needing to actively search for information), it is imperative that the information given to students is comprehensive enough that follow-up independent research is not required (although still signposted).

### University

Many women feel that university is an appropriate place to deliver menopause education; women under 20 were most likely to feel this way with 61.0% of them selecting this option. The importance of higher education environments delivering health education to its students is acknowledged by the WHO, and a report titled ‘Health Promoting Universities’ suggests ways in which this can be done.^26^ The main limitation of delivering menopause education in a university setting is that not all young people attend higher education; however, it is becoming an increasingly popular choice in the United Kingdom; in 2021 37.9% of 18-year-olds went to university.^27^ This growth in attendance demonstrates that university could become an extremely effective way to educate a significant proportion of young people on the menopause.

### In the workplace

As women under 40 were not educated on the menopause at school providing alternative education sources later in life is essential. The delivery of menopause education in the workplace was suggested by multiple women under 40, particularly those aged 21–30 or over 30. The fact that young people spend most of their time at work^28^ reiterates how valuable the workplace could become to deliver effective menopause education. The WHO^29^ and Centers for Disease Control and Prevention^30^ emphasize the importance of the workplace as an environment for health education, and studies have shown that education programmes in the workplace are effective.^31,32^ Data from the government report ‘Menopause transition: effects on women’s economic participation’ showed that nearly 8 out of 10 menopausal women are in work.^33^ British United Provident Association Limited (BUPA) also reported that three out of five of menopausal women were negatively affected at work.^34^ These figures provide further evidence that delivering menopause education in the workplace is essential.

The benefits of menopause education in the workplace were highlighted by multiple women in our study; many feel it would reduce the ‘taboo’ attached to the menopause in the workplace and help to normalize the menopausal transition. The Royal College of Obstetricians and Gynaecologists’ (RCOG)^35^ ‘Better Health for Women’ report reiterates this, recognizing the opportunity menopause education in the workplace provides to reduce stigma and keep women in the workplace.

Our survey outlines one method that could be used to deliver menopause education in the workplace – via online courses. One participant had partaken in learning by this format through the workplace and found it to be an effective way to learn about the menopause. Although many working environments use E-learning and find it effective,^36^ the majority do not currently offer education on the menopause via this method (or at all). The Chartered Institute of Personnel and Development^37^ and Acas^38^ provide advice to employers on how they can support women going through the menopause at work, and the government acknowledges that an integrated approach to menopause education in the workplace is essential.^33^ Our study recommends that further research is conducted assessing the efficacy of different learning formats for health education in the workplace (and beyond). Through assessing this, and ultimately establishing an effective technique for menopause education in the workplace, we can increase the knowledge of both women under 40, and wider society, about the menopause significantly.

### GP education

Education of the menopause among healthcare professionals is also sparse. General practitioners (GPs) lack both knowledge and confidence when recognizing symptoms of the menopause^5^ and prescribing appropriate management.^39,40^ This issue starts right at the beginning of medical training; 41% of UK medical schools do not have mandatory menopause education.^41^ The BMS^42^ aims to improve the knowledge of healthcare professionals by providing information and guidance. All GPs should be trained to recognize menopause symptoms as this was one of the largest hurdles women reported. Understanding treatment options can be highly specialized and may be best suited to a GP with more advanced training.

### Public health campaigns and government action

A further suggestion made by women under 40 was to deliver menopause education via public health campaigns. One participant’s suggestion was that menopause information is distributed in a similar way to how the government promotes a ‘healthy diet’. Our finding that many young women have acquired their knowledge on the menopause fairly passively reiterates why learning in this format could be effective; public health campaigns support passive and incidental learning by stimulating increased discussion of the topic in society. Furthermore, we found that many women think menopause resources are generally unreliable and difficult to access; public health campaigns overcome both these issues. The WHO^43^ recognizes the importance of public health campaigns to promote health education, and research has shown that previous campaigns have been effective; the governments ‘change4life’ campaign had significant benefits for child health.^44^ Further studies have reiterated these benefits, finding that campaigns improve both awareness and knowledge around health issues;^45^ however, research into direct behaviour change is limited.^46^ Currently, in the United Kingdom there is no official public health campaign around the menopause, but the government accepts that menopause education must be prioritized moving forward; the 2019 Sex and Relations Education Regulations is one example of this.^4^ Further evidence of this acceptance is the government’s ‘Women’s Health Strategy’, where goals set include education as a key thematic priority and menopause as a key priority area.^12^ To fulfil these goals the UK Menopause Taskforce has been established, a committee that aims to destigmatise and improve education of the menopause.^47^ Our study, alongside the fact that menopause is an essential public health dilemma,^48^ demonstrates that a public health campaign would be an appropriate next step for the government to take. This will ultimately increase societal knowledge and reduce the stigma associated with the menopause.

### Social media and apps

Our data show that less than 40% of women under 40, across all age groups, currently use social media to learn about the menopause. We also found that most young women learn about the menopause fairly passively, interacting with the topic incidentally. In recognizing these findings, alongside the statistic that 79% of the United Kingdom use social media,^49^ it is clear that social media has the potential to become a far more powerful menopause education source for women under 40. Distributing information on the menopause via social media platforms that women are likely already using, will increase their interaction with the topic and provide more learning opportunities without them needing to be proactive. By doing so, menopause education via social media supports passive and incidental learning and should stimulate a more open discussion and lifelong interest in the menopause for young women. In 2017, WHO^50^ described the intersection between electronic health and public health as a ‘beautiful interaction’, reflecting the growing acceptance that the Internet is important in providing public health education. Social media is recognized as a particularly valuable tool within this sphere due to its high public engagement (4.62 billion users^51^) and ability to remove physical barriers that often prevent access to information.^52^ COVID-19 information was distributed effectively on social media during the pandemic, mainly via TikTok,^53^ and thus it can be recommended that menopause information is distributed in a similar fashion. Studies have also shown that public health campaigns are most effective when they target individuals based on sociodemographic characteristics.^54^ Social media platforms provide an opportunity, via their algorithm, to distribute campaigns to their users according to these characteristics. This is further evidence that social media could become an even more powerful tool to educate future generations on the menopause, and one we must embrace.

Resources to support women through the menopause are increasing, with information available through websites such as ‘Menopause and Me’,^55^ menopause matters,^56^ Women’s Health Concern^57^ and apps like ‘Balance’.^58^ However, not all these platforms are well sourced; only 27.3% of menopause apps available had medical professionals included in their development.^59^ A separate concern is that not all sources are thorough. With such a limited list of symptoms, it is concerning that even if women take the initiative to search for their symptom online, they may not be able to link it with the menopause. More globally, an important issue is that many menopause support resources reside online. Although this is not hugely problematic in the United Kingdom where access to the Internet is commonplace, in cultures and locations where there is limited Internet access this is a significant barrier to menopause education.^60^

Apps such as fertility and period trackers were recognized as an important method of delivering menopause education by 70% of women aged 21–30 and under 20. Studies have shown the benefit of apps for learning, revealing that they can greatly impact health behaviours^61^ and enhance outcomes for users.^62^ Furthermore, they are both cost-effective and easy to access for women.^63^ As of 2019, there were 22 apps related to the menopause available to download.^59^ However, although apps are available, a study has shown that only 22.7% contain evidence-based information and just 27.3% had medical professionals involved in their development.^59^ Considering our findings that women under 40 value apps to learn about the menopause, it is essential that there is greater input from medical professionals when developing and publishing information on these apps in future. This will ultimately make apps a more valid and reliable source from which women can learn about the menopause.

## Limitations

It is essential to acknowledge that there are limitations of this study. The first of these is using an online survey to collect our data. The primary issue with online surveys is that it is impossible to ensure that the survey was only answered by women that fulfil our inclusion criteria. As a result of this, our results might be less authentic and/or misrepresent the target population.^64^ The women were only English speaking, and few ethnicities were included. Although this is a concern, due to our desire to collect a significant number of responses, we decided to proceed with the online format and rely on the candour of participants to mitigate this risk. A further issue with online surveys is the risk of self-selection bias. Naturally, there is a tendency that some women will ignore the survey while others will proactively complete it, thus admitting some systematic bias.^65^

There are also limitations of using social media to distribute our survey. First, unless the survey is sent directly to them, we eliminate anyone who does not use social media from participating.^66^ Furthermore, even in the population with social media, those that are more engaged on the platforms (usually the younger generation) are more likely to see and complete the survey. To this effect, demographic sampling bias may be admitted,^67^ and our participant cohort might more heavily weighted towards young, highly educated women. The fact our cohort was comprised of women mainly of White-UK ethnicity outlines a further limitation of this study. Future research focusing on a more ethnically and socioeconomically diverse population is essential, and we are currently running menopause surveys specifically for Black and Asian women. It is also essential to recognize that when using social media to distribute our survey we relied on the algorithms of these platforms to promote our posts to users. These algorithms are complex, often targeting certain demographics with particular posts and thus, this may admit further bias. Using a paid advert on Twitter gave us some control over this algorithm as we could select a particular demographic to target with our survey.

## Conclusion

This study highlights that most women under 40 have little to no knowledge surrounding the menopause. The limited knowledge women do have has been acquired via self-directed learning as opposed to formal education sources such as school. Differences in the way women conducted this self-directed learning were observed between age groups. Women under 20 took a more passive approach to learning, mainly using family, while women over 30 were more proactive, using official websites and scientific literature. Application of these findings can help to direct menopause education moving forward.

To ensure education of the menopause in schools is effective, it must be thorough and delivered to both all pupils. Beyond school, a multifaceted approach to menopause education is advised. It is recommended that menopause education is delivered in the workplace, via social media and through public health campaigns. It is only with a combination of these approaches that menopause education can be effectively delivered throughout the life course.

## Supplemental Material

sj-docx-1-whe-10.1177_17455057221139660 – Supplemental material for Menopause knowledge and education in women under 40: Results from an online survey Click here for additional data file. Supplemental material, sj-docx-1-whe-10.1177_17455057221139660 for Menopause knowledge and education in women under 40: Results from an online survey by Carly Munn, Leigh Vaughan, Vikram Talaulikar, Melanie C Davies and Joyce C Harper in Women’s Health

sj-docx-2-whe-10.1177_17455057221139660 – Supplemental material for Menopause knowledge and education in women under 40: Results from an online survey Click here for additional data file. Supplemental material, sj-docx-2-whe-10.1177_17455057221139660 for Menopause knowledge and education in women under 40: Results from an online survey by Carly Munn, Leigh Vaughan, Vikram Talaulikar, Melanie C Davies and Joyce C Harper in Women’s Health

## Declarations

**Ethics approval and consent to participate:** The survey was approved by the UCL Research Ethics Committee ID no: 9831/005. Patient consent was embedded at the start of the survey and had to be completed before progressing.

**Consent for publication:** Not applicable.

**Availability of data and materials:** Because of ethical approval, the data cannot be widely available.
