# Supplementary material for: Menopause knowledge and education in women under 40: Results from an online survey
Source: Womens Health (Lond). 2022 Dec 19;18:17455057221139660. doi: 10.1177/17455057221139660 (PMC9772977; doi:10.1177/17455057221139660)
Supplement: sj-docx-1-whe-10.1177_17455057221139660 – Supplemental material for Menopause knowledge and education in women under 40: Results from an online survey [file sj-docx-1-whe-10.1177_17455057221139660.docx]

**Supplementary Data**

**Survey**

Evaluation of how women under 40 learn, and their attitudes to, the menopause

Start of Block: Consent

Q1 **UCL ethics committee approval: 9831/005**   **Title of Study: Evaluation of how women under 40 learn, and their attitudes to, the menopause.**   **Department: UCL EGA Institute for Women’s Health**   **Name and Contact Details of the Principal Researcher:**Professor Joyce Harper.  Institute for Women’s Health, University College London. Email: joyce.harper@ucl.ac.uk Telephone: 07880 795791    **1.     Introduction.** You have been invited to take part in a University College London, Institute for Women’s Health research study being conducted to evaluate how women under 40 learn about the perimenopause and menopause. This survey is for women who have not gone through an early menopause. Before you decide it is important for you to understand why the research is being done and what participation will involve. Please take time to read the following information carefully and discuss it with others if you wish.  Ask us if there is anything that is not clear or if you would like more information. Take time to decide whether or not you wish to take part. Thank you for reading this.       **2. What is the project’s purpose?**   All women will go through the menopause but menopause education is almost non-existent.  As a result, very few women know about the common symptoms of the menopause and many will suffer peri/menopausal symptoms for a number of years without realising the cause.  In this survey we aim to understand more about when and how women under 40 are learning about the peri/menopause with the aim of improving peri/menopause education.  As is routine when doing surveys, we will ask you some questions about your demographics, such as your religion, ethnicity, sexual orientation, and age so we can determine the type of women who have completed the survey.    **3. Do I have to take part?**  It is up to you to decide whether or not to take part.  If you do decide to take part you will be asked to answer the questions in the online survey.  You can withdraw at any time without giving a reason – simply do not submit your answers.  If you decide to withdraw before completing the survey, your answers will not be used in the study.  Once you have submitted your answers, we cannot withdraw them as the survey is anonymous.     **4. What are the possible disadvantages and risks of taking part?** The UCL Research Ethics Committee have approved this study, and we do not anticipate any risks to any individuals taking part in this study.  After completing the survey, we will direct you to the menopause poster which has additional information. If you have any concerns about the answers to the questions in this survey relating to your health, you may wish to contact your doctor.   **5. What are the possible benefits of taking part?** You may learn some information about the menopause.   **6. What if something goes wrong?**  If you have any complaints regarding your treatment by the researchers, you can complain to Professor Harper on joyce.harper@ucl.ac.uk or to the Chair of UCL Ethics.  In the unlikely event of something serious occurring during or following your participation in the project, please also contact Professor Harper.  However should you feel your complaint has not been handled to your satisfaction please contact the Chair of the UCL Research Ethics Committee – ethics@ucl.ac.uk.             **7. Will my taking part in this project be kept confidential?** All the information that we collect about you during the course of the research will be kept strictly confidential.  You will not be able to be identified in any ensuing reports or publications.  
    **8. What will happen to the results of the research project?** Following completion of the study, we aim to publish the results in a peer-reviewed journal and present the data at conferences and on social media.  The anonymised data may be used by others for future research but no one will be able to identify you when this data is shared.   **9. Local Data Protection Privacy Notice.** The controller for this project will be University College London (UCL). The UCL Data Protection Officer provides oversight of UCL activities involving the processing of personal data task in the public interest, and can be contacted at data-protection@ucl.ac.uk. This ‘local’ privacy notice sets out the information that applies to this particular study. Further information on how UCL uses participant information can be found in our ‘general’ privacy notice:  The information that is required to be provided to participants under data protection legislation (GDPR and DPA 2018) is provided across both the ‘local’ and ‘general’ privacy notices.     Your personal data will be processed so long as it is required for the research project. If you are concerned about how your personal data is being processed, or if you would like to contact us about your rights, please contact UCL in the first instance at data-protection@ucl.ac.uk.     UCL’s Data Protection Officer is Alex Potts - data-protection@ucl.ac.uk        Thank you for reading this information sheet and for considering to take part in this research study. Your consent is important to us.  Please can you click on the consent button below.   

Q2 **Please read carefully:**
   
  I have read the above information page and understand what the study involves and  ·       I understand that if I decide at any time that I no longer wish to take part in this project, I can withdraw immediately by not submitting my answers.  But once I have submitted, I cannot withdraw.  ·       consent to the processing of my anonymised personal information for the purposes of this research study.  ·       understand that such information will be treated as strictly confidential and handled in accordance with the provisions of the Data Protection Act 1998.  ·       agree that the research project named above has been explained to me to my satisfaction and I agree to take part in this study.  ·      agree that my data, which is fully anonymised, can be shared with other researchers. 
 Am I eligible to take part in this survey? 
> I am female 
> I am aged 40 or under (if you are over 40 please do not complete this survey)
> I have not gone through an early menopause
 If you answered yes to the questions above, and are able to read and speak English, you will be eligible to take part in this study.       **Consent: I confirm that I am a female aged 40 or under.  I have read and agree with the statements above**

- Consent (1)
- I do not consent (2)

Q3   **There are three parts to the survey. We would like to ask you about yourself, your experience of the menopause and learning about the menopause and finally some basic information about your background.  It is really important that you complete all three parts as incomplete surveys cannot be included in our analysis. You will know when your survey results are submitted as you will receive a copy of the menopause poster. Thank you.**

End of Block: Consent

Start of Block: Default Question Block

| Page Break |  |
| --- | --- |

Q4 **Please state your country of residence**

- UK (4)
- Other - please state (5) ________________________________________________

| 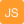 |
| --- |

Q5 **Please state your age in years:**

________________________________________________________________

Q6 **Please state your sexual orientation.**

- Heterosexual (1)
- Homosexual (2)
- Bisexual (3)
- Pansexual (4)
- Asexual (5)
- Prefer not to say (6)

Q7 **Please select the option that most reflects your current relationship status.**

- Single (1)
- In a relationship not cohabiting (2)
- In a relationship cohabiting (3)
- Married/civil partnership (4)
- Widowed (9)
- Prefer not to say (7)
- Other - in your own words (8) ________________________________________________

Q8 **Do you have children?**

- 1 (1)
- 2 (2)
- 3 (3)
- 4 or more (4)
- I do not have children (5)
- Prefer not to say (6)

| 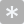 |
| --- |

Q9 **Relating to your wellbeing, tick all that apply.**

- I regularly exercise (1)
- I sleep well (2)
- I eat a healthy diet most of the time/always (3)
- I am a moderate alcohol drinker (4)
- I do not drink alcohol (9)
- I do not smoke (5)
- I have good mental health (6)
- None of the above (8)
- Prefer not to say (7)

Q10 **Are you currently using any methods below as a form of contraception (do not tick if using for other reasons apart from contraception, such as for heavy periods).**

- Female condom (1)
- Male condom (2)
- Combined pill (3)
- Progesterone only (mini) pill (4)
- Contraceptive Implant (5)
- Contraceptive Injection (6)
- Contraceptive Patch (7)
- Vaginal Ring (8)
- Diaphragm (9)
- Mirena coil - Intrauterine system (IUS) (10)
- Copper coil - Intrauterine device (IUD) (11)
- Fertility awareness methods (body temperature ovulation sticks) with an app (12)
- Fertility awareness methods (body temperature ovulation sticks) without an app (13)
- Withdrawal method (14)
- Male sterilisation (15)
- Female sterilisation (16)
- None of the above (19)

Q11   **Part 2: We would like to ask you about your learning of the perimenopause/menopause, your experience and your attitude.**

Q12 **How do you feel about the peri-menopause/menopause?**

- Not looking forward to it (9)
- Neutral - no strong view either way (8)
- Accepting of it (7)
- Looking forward to it (6)
- Have not thought about it (11)
- Not sure (10)

Q13 **What are your thoughts about no longer having periods?**

- Happy (1)
- Neutral - no strong view either way (2)
- Will miss not having a period (7)
- Not thought about it (5)
- Other - Please tell us more in your own words (4) ________________________________________________

Q14 **Where do you think the menopause should be taught? Tick all that apply.**

- School (5)
- University (8)
- Doctor's surgery (9)
- Contraception clinic (10)
- Apps such as period trackers and fertility apps (11)
- Pregnancy clinic (12)
- Other - please give details (7) ________________________________________________

Q15 **How were you taught about the menopause at school?**

- Very detailed (1)
- Basic (2)
- Not at all (3)

Q16 **How informed do you feel about perimenopause/menopause?**

- Very informed (1)
- Some knowledge (2)
- Not informed at all (3)
- Not sure (4)

Q17 Have you looked for information or discussed the menopause?

- Yes (1)
- No (2)
- Not sure (3)

Skip To: Q21 If Have you looked for information or discussed the menopause? = No

Q18 **What age were you when you looked for information/discussed the menopause?**

- Please put the age when you started thinking about the perimenopause/menopause (10) ________________________________________________
- I cannot remember (11)

Q19 Why did you look for information about the menopause?

- Someone I know was going through it (1)
- I thought i was having symptoms (2)
- Heard about it in the news/socialmedia/media (3)
- Was interested (4)
- Other (5) ________________________________________________

Q20   **Have you specifically looked for information of the menopause in any of these ways – tick all that apply**

- Official web sites such as the menopause society (14)
- Other web sites (1)
- YouTube (2)
- Podcasts (13)
- Social media (11)
- Magazines (3)
- Newspapers (4)
- Books (5)
- Documentaries (6)
- Films and TV programs (7)
- Family (15)
- Friends (8)
- Health professionals (9)
- Scientific literature (10)
- Have heard those around me discussing it (16)
- Other - please give details (12) ________________________________________________

Q21 **Do you feel there is an open discussion with friends/family about the menopause?**

- We often talk about it (2)
- We sometimes talk about it (1)
- We feel able to talk about it when it comes up (4)
- We never talk about it (6)
- It has never come up (5)

| 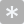 |
| --- |

Q22   **Which of the following do you think are perimenopausal/menopausal symptoms? Tick all that apply:**

- Hot flushes (12)
- Night sweats (1)
- Irregular periods (2)
- Heavy periods (32)
- Painful periods (33)
- Mood swings (7)
- Depression (27)
- Anxiety (21)
- Paranoia (23)
- Brain fog (30)
- Difficulty/poor concentration (46)
- Headaches/migraines (19)
- Poor memory (14)
- Tearful (36)
- Irritability (58)
- Low mood (31)
- Reduced confidence (54)
- Dizziness (15)
- Insomnia/problems sleeping (20)
- Fatigue (8)
- Lack of motivation (53)
- Low sex drive (45)
- Loss of sex drive (3)
- Vaginal dryness (4)
- Vaginal problems (5)
- Itching - ears, body, vagina, anywhere (6)
- Tingling in hands/arms/legs/feet (29)
- Clammy feeling (44)
- Heart palpitations (24)
- Weight gain (16)
- Bloating (41)
- Digestive issues (48)
- Osteoporosis (35)
- Aching joints (17)
- Aching muscles (18)
- Muscle tension (47)
- Restless leg syndrome (28)
- Burning tongue/roof of mouth (25)
- Gum problems (40)
- Bad Breath (51)
- Ears ringing (tinnitus) (52)
- Facial hair growth (49)
- Hair loss/thinning (59)
- Body odour (39)
- Incontinence (37)
- Urinary symptoms (55)
- Breast soreness (38)
- Weak nails (57)
- Brittle nails (42)
- Cold Flushes (43)
- Electric shock sensations (50)
- Increased allergies (56)
- Other - please state in your own words (34) ________________________________________________

Q23 **Have you spoken with a health professional about the peri/menopause?**

- Yes (1)
- No (2)
- Aiming to soon (3)

Q24 **Do you know which of the following can be used to alleviate perimenopausal/menopausal symptoms? Tick all that apply.**

- Nutrition changes including reduced alcohol/caffeine (1)
- Exercise (11)
- Hormone replacement therapy (HRT) (2)
- Bio identical hormones (6)
- Compounded hormones (7)
- Mirena coil (IUS) (8)
- Topical oestrogen (9)
- Cognitive behavioural therapy (12)
- Serotonin uptake inhibitors (13)
- Complementary therapies such as homeopathy, acupuncture, etc - please give details (5) ________________________________________________
- Other - please give details (4) ________________________________________________
- I have not heard of any of these methods (25)

Q25 **In your own words, would you like to tell us anything about your views of the peri-menopause/menopause?**

________________________________________________________________

________________________________________________________________

________________________________________________________________

________________________________________________________________

________________________________________________________________

Q26     **Part 3: Finally, we need to know a little about your background so we can compare different groups of people.  It is really important that you complete this section, as otherwise we cannot use your answers. You will know when the survey is submitted as you will receive a copy of the menopause poster.**

| Page Break |  |
| --- | --- |

Q27 **What is your highest educational qualification?**

- Secondary School (1)
- A Level/College-level (6)
- University undergraduate (2)
- University postgraduate (3)
- Other (4) ________________________________________________
- Prefer not to say (5)

Q28 **What is your profession?**

________________________________________________________________

Q29 **What is your religion or belief:**

- No religion or belief (1)
- Christian including Church of England, Catholic, Protestant and all other Christian denominations (2)
- Hindu (3)
- Jewish (4)
- Muslim (5)
- Sikh (6)
- Buddhist (7)
- Any other religion or belief - please give details (8) ________________________________________________
- Prefer not to say (9)

Q30 **How do you identify yourself?  Choose one or more.**

- White - English / Welsh / Scottish / Northern Irish / British (1)
- White - Irish (2)
- Any other White background (please specify) (3) ________________________________________________
- Black/Black British - African (4)
- Black/Black British - Caribbean (10)
- Any other Black/African/Caribbean background (please specify) (11) ________________________________________________
- Latino (16)
- Asian/Asian British - Indian (12)
- Asian/Asian British - Pakistani (13)
- Any other Asian background (please specify) (5) ________________________________________________
- Arab (8)
- Mixed ethnic background (please specify) (7) ________________________________________________
- Any other ethnic group, please describe (9) ________________________________________________
- Prefer not to say (15)

Q31 **What is your disability status?** The Equality Act 2010 states a person has a disability if they have a physical or mental impairment that has a substantial and long-term adverse effect (likely to last 12 months or more) on their ability to perform normal day-to-day activities (e.g. eating, washing, walking and going shopping).

- No disability (1)
- Sensory impaired (2)
- Physical or mobility impaired (3)
- Specific learning difficulty or disability (e.g. dyslexia) (4)
- General learning disability (cognitive) (5)
- Long term illness or health condition (6)
- Autistic spectrum disorder (7)
- Other, please specify (8) ________________________________________________
- Prefer not to say (9)

| Page Break |  |
| --- | --- |
